# Overlapping Delta and Omicron Outbreaks During the COVID-19 Pandemic: Dynamic Panel Data Estimates

**DOI:** 10.2196/37377

**Published:** 2022-06-03

**Authors:** Alexander L Lundberg, Ramon Lorenzo-Redondo, Judd F Hultquist, Claudia A Hawkins, Egon A Ozer, Sarah B Welch, P V Vara Prasad, Chad J Achenbach, Janine I White, James F Oehmke, Robert L Murphy, Robert J Havey, Lori A Post

**Affiliations:** 1 Buehler Center for Health Policy and Economics Robert J Havey, MD Institute for Global Health Northwestern University Chicago, IL United States; 2 Department of Emergency Medicine Feinberg School of Medicine Northwestern University Chicago, IL United States; 3 Department of Medicine Division of Infectious Diseases Feinberg School of Medicine, Northwestern University Chicago, IL United States; 4 Center for Pathogen Genomics and Microbial Evolution Robert J Havey, MD Institute for Global Health Northwestern University Chicago, IL United States; 5 Center for Global Communicable and Emerging Infectious Diseases Robert J Havey, MD Institute for Global Health Northwestern University Chicago, IL United States; 6 Sustainable Intensification Innovation Lab Kansas State University Manhattan, KS United States; 7 Robert J Havey, MD Institute for Global Health Feinberg School of Medicine Northwestern University Chicago, IL United States; 8 Department of Medicine, General Internal Medicine and Geriatrics Feinberg School of Medicine Northwestern University Chicago, IL United States

**Keywords:** Omicron variant of concern, Delta, COVID-19, SARS-CoV-2, B.1.1.529, outbreak, Arellano-Bond estimator, dynamic panel data, stringency index, surveillance, disease transmission metrics

## Abstract

**Background:**

The Omicron variant of SARS-CoV-2 is more transmissible than prior variants of concern (VOCs). It has caused the largest outbreaks in the pandemic, with increases in mortality and hospitalizations. Early data on the spread of Omicron were captured in countries with relatively low case counts, so it was unclear how the arrival of Omicron would impact the trajectory of the pandemic in countries already experiencing high levels of community transmission of Delta.

**Objective:**

The objective of this study is to quantify and explain the impact of Omicron on pandemic trajectories and how they differ between countries that were or were not in a Delta outbreak at the time Omicron occurred.

**Methods:**

We used SARS-CoV-2 surveillance and genetic sequence data to classify countries into 2 groups: those that were in a Delta outbreak (defined by at least 10 novel daily transmissions per 100,000 population) when Omicron was first sequenced in the country and those that were not. We used trend analysis, survival curves, and dynamic panel regression models to compare outbreaks in the 2 groups over the period from November 1, 2021, to February 11, 2022. We summarized the outbreaks in terms of their peak rate of SARS-CoV-2 infections and the duration of time the outbreaks took to reach the peak rate.

**Results:**

Countries that were already in an outbreak with predominantly Delta lineages when Omicron arrived took longer to reach their peak rate and saw greater than a twofold increase (2.04) in the average apex of the Omicron outbreak compared to countries that were not yet in an outbreak.

**Conclusions:**

These results suggest that high community transmission of Delta at the time of the first detection of Omicron was not protective, but rather preluded larger outbreaks in those countries. Outbreak status may reflect a generally susceptible population, due to overlapping factors, including climate, policy, and individual behavior. In the absence of strong mitigation measures, arrival of a new, more transmissible variant in these countries is therefore more likely to lead to larger outbreaks. Alternately, countries with enhanced surveillance programs and incentives may be more likely to both exist in an outbreak status and detect more cases during an outbreak, resulting in a spurious relationship. Either way, these data argue against herd immunity mitigating future outbreaks with variants that have undergone significant antigenic shifts.

## Introduction

### Background

Omicron, or B.1.1.529, the latest SARS-CoV-2 variant of concern (VOC), was first sequenced in Botswana in early November 2021 [1]. South Africa reported Omicron to the World Health Organization (WHO) on November 24, 2021, and the WHO designated it as a VOC on November 26, 2021 [2,3]. Early reports of Omicron from South Africa alarmed infectious disease scientists due to both its rapid spread in the population and the high degree of molecular divergence in the spike protein [4,5]. Omicron spread quickly through South Africa’s population despite serological evidence of prior SARS-CoV-2 infections or vaccinations in 60%-80% of its population [6]. Omicron was better able to evade natural and vaccine-induced immunity compared to previous variants [7,8]. Ultimately, it was found to be less severe in terms of infection and symptoms than other VOCs [9], especially for those persons who received 2 messenger RNA (mRNA) vaccines and a booster [10]. However, estimated vaccine effectiveness in terms of transmissions was still lower against Omicron compared to Delta [11]. Full mRNA vaccinations plus booster caused a 70% reduction in deaths and hospitalizations compared to no vaccine [12-14]. With higher transmissibility, case counts began setting daily records [15] and health systems were overwhelmed as the Omicron VOC spread SARS-CoV-2 [2,16].

Omicron shifted the course of the pandemic because of its increased transmissibility and its relatively enhanced ability to evade immunity from vaccination or prior infection [17,18]. Early research demonstrated that the Omicron VOC gave fewer days of warning leading up to an outbreak compared to Delta, Alpha, Beta, and the original reference strain (D614) [19]. By early 2022, it was evident that Omicron was setting 2-year record highs in the number of daily new transmissions, displacing Delta as the most transmissible VOC [19]. Moreover, Omicron infections had a significant growth advantage over Delta, with a doubling period of new cases of 1.5-3 days [20,21]. The magnitude of the outbreak, as measured by its apex, was 1.5 to 2-fold higher than prior outbreaks [19].

Early observations in sub-Saharan Africa showed a consistent trend where Omicron cases quickly accelerated, and then quickly decelerated after peaking with only a slight tail [19]. However, these countries had relatively low cases counts prior to the arrival of Omicron. As Omicron spread to countries already experiencing high community transmission of Delta, it was unclear whether the trajectory of the outbreak would be altered. On the one hand, policy mitigation efforts put in place to combat ongoing outbreaks, combined with a higher frequency of natural immunity in the population, could reduce the magnitude of a subsequent outbreak [22]. On the other hand, a preexisting outbreak may signal underlying factors (stringency of mitigation measures, weather, etc) that are favorable to larger outbreaks with more transmissible variants [23-25]. In this study, we compared the trajectories/trends in the Omicron outbreak between countries that had low levels of community transmission of Delta and those that were already in a Delta-driven outbreak [19].

### Objective

The objective of this study is to quantify and explain the impact of Omicron on pandemic trajectories and how they differ between countries that were or were not in a SARS-CoV-2 outbreak with Delta at the time Omicron arrived.

## Methods

### Data Collection

We used SARS-CoV-2 surveillance data to identify the duration and apex of outbreaks [26] and GISAID (Global Initiative on Sharing Avian Influenza Data) to identify VOCs [27]. We modeled the data using trend analysis [28-37], survival curves [38,39], and dynamic panel regression [40,41]. We conducted the analysis in R version 4.1.1 (R Foundation for Statistical Computing) with the *plm* (version 2.4-1), *survival* (version 3.2-13), and *survminer* (version 0.4.9) packages [42-45]. The sample period covered November 1, 2021, to February 11, 2022.

To estimate the date Omicron first appeared in a country, we used publicly available data on sequenced SARS-CoV-2 variants from GISAID [27]. We used Nextclade nomenclature [46] to collect clade designations from sequences and Pangolin nomenclature for lineage designations of SARS-CoV-2 [47,48]. We also contrasted prevalence data with data compiled from outbreak.info [49]. We classified countries into 2 groups: (1) outbreak countries that exceeded a threshold of 10 novel daily SARS-CoV-2 transmissions per 100,000 population at the onset date of Omicron, defined by the first instance of an Omicron clade in GISAID, and (2) nonoutbreak countries below this transmission threshold at the first instance of an Omicron clade. The outbreak threshold follows the convention adopted by the US Centers for Disease Control and Prevention (CDC).

To maximize comparability across outbreaks, we restricted the sample to dates between November 1, 2021, and February 11, 2022. Within this period, surveillance sequencing in all countries consisted predominantly of Delta and Omicron variants, with all other variants comprising less than 0.03% of total sequences.

We excluded island countries with populations below a half million people because their outbreaks follow distinct trajectories [40]. Sequencing data are not available for every country [27], so our sample was restricted to 80 countries. Of them, 42 (52.5%) were already in an outbreak at the onset of Omicron, and 38 (47.5%) were not.

### Statistical Analysis

We estimated Kaplan-Meier survival curves for the outbreak and nonoutbreak groups to compare the length of time a country takes to reach its apex speed after the onset of Omicron [39]. We consider any country whose apex speed occurred on the final date of the sample to be censored. We also provided a trend comparison for several neighbor countries, at least 1 of whom was in the outbreak group and 1 of whom was not.

We used linear trend analysis to compare apex speed across the outbreak and nonoutbreak groups. To control for differences in population vaccination rates, prior infection rates, time since the onset of Omicron, and any time-invariant, country-specific heterogeneity, we estimated a dynamic panel regression model with the Arellano-Bond method [40,41]. We modeled the daily rate of novel transmissions as a function of 1-day and 1-week lagged transmissions, cumulative infection and vaccination rates, a binary weekend indicator, the number of days since the onset of Omicron, the number of days since a Delta outbreak began, a binary indicator for whether the date is after the earliest sequenced Omicron variant isolated in the country, and an interaction between the latter and an indicator for whether the country was in the outbreak group. The interaction term provides a test for whether Omicron generated larger increases in speed for the outbreak countries versus the nonoutbreak countries.

We tested the possibility of a weather-driven spurious effect that resulted in different outbreak trajectories with an extension of the survival analysis. We compared Kaplan-Meier survival curves for outbreaks in countries in the Northern and Southern Hemispheres. To address the possibility of SARS-CoV-2 policy response as a confounder, we calculated the average stringency index for each country over the sample period [50]. We conducted a Welch *t* test to compare the average index score in countries in the outbreak versus nonoutbreak groups.

## Results

### Omicron and Outbreak Trajectory

**Figure 1:**
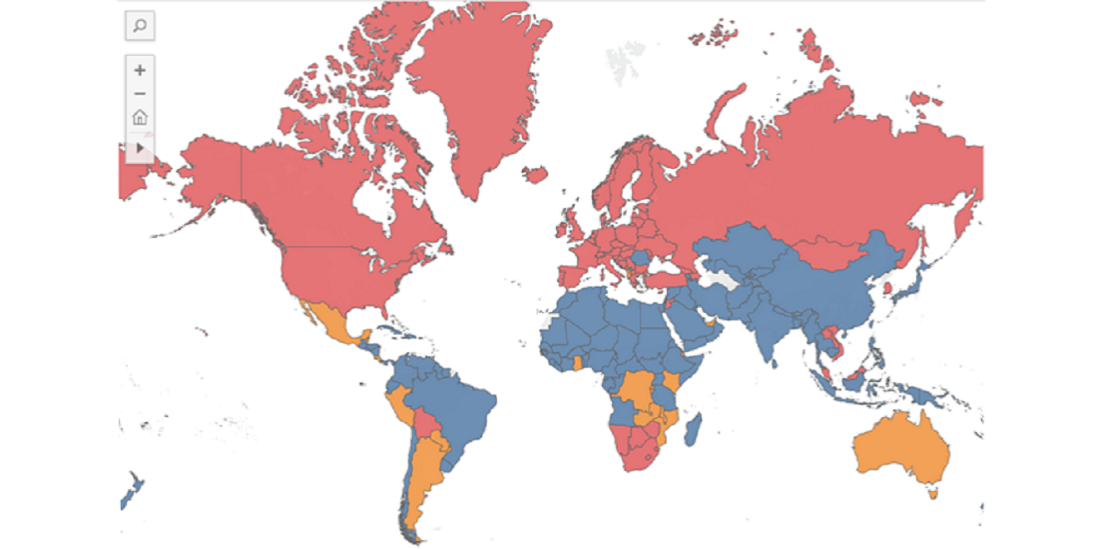
Map of Delta and Omicron outbreaks. Note that countries in red were in an outbreak on December 15, 2022, as defined by a daily rate of at least 10 novel SARS-CoV-2 transmissions per 100,000 population. Countries in orange were not in an outbreak but displayed 7 consecutive days of an increase in the rate of novel SARS-CoV-2 transmissions per 100,000 population.

Figure 1 is a map of SARS-CoV-2 outbreaks on December 15, 2022. At this point in time, we can see countries already in a Delta outbreak, countries in an Omicron outbreak in the South of sub-Saharan Africa, and countries with exponential growth due to the introduction of Omicron to the genetic pool. Countries in blue are not in an outbreak. Countries in orange are not in an outbreak but are experiencing alarming growth across 7 consecutive days that will likely go into an outbreak if left unabated. Countries in red in North America, Europe, Central Asia, and some of East Asia and the Pacific are already in an outbreak, primarily driven by Delta.

Table 1 presents both speed (or the number of daily new SARS-CoV-2 transmissions per 100,000 population) at the onset of Omicron and peak speed for all countries in the sample. The countries experiencing high levels of community transmission of Delta at the time of Omicron’s arrival (“already in outbreak”) averaged a speed of 52.6 at the onset of Omicron, while the countries experiencing low levels of transmission at the onset of Omicron (“not in outbreak”) averaged a speed of 3.2. The respective average peak speeds were 308.7 and 128.6. Thus, even after controlling for the initial differences in speed, the countries already in an outbreak saw greater than a twofold increase (2.04=[308.7 – 52.6]/[128.6 – 3.2]) in the average apex of an outbreak.

**Table 1:** Outbreak status when index Omicron case sequenced.

Country		Speed at Omicron		Peak speed		Average speed		Average peak speed	
**Already in outbreak**							52.6		308.7
	Austria		154.5		374.8	N/A^a^		N/A	
	Belgium		119.3		449.9	N/A		N/A	
	Bosnia and Herzegovina		13.1		73.9	N/A		N/A	
	Botswana		39		63.9	N/A		N/A	
	Bulgaria		18.7		128.7	N/A		N/A	
	Chile		12.4		186.2	N/A		N/A	
	Croatia		108.4		217	N/A		N/A	
	Czech Republic		85.2		355.5	N/A		N/A	
	Denmark		67.1		807.5	N/A		N/A	
	Estonia		38.4		520.9	N/A		N/A	
	Finland		19.8		152.3	N/A		N/A	
	France		13.5		562.3	N/A		N/A	
	Georgia		83.2		543.7	N/A		N/A	
	Germany		58.7		231.4	N/A		N/A	
	Greece		61.6		347.6	N/A		N/A	
	Ireland		90.3		481	N/A		N/A	
	Italy		15.8		300.7	N/A		N/A	
	Jordan		47.9		192.9	N/A		N/A	
	Lebanon		22		121	N/A		N/A	
	Liechtenstein		141.3		380.7	N/A		N/A	
	Lithuania		64.5		402.5	N/A		N/A	
	Luxembourg		61.6		369.9	N/A		N/A	
	Malaysia		17.9		44.7	N/A		N/A	
	Malta		19.7		258.8	N/A		N/A	
	Montenegro		38.1		393.6	N/A		N/A	
	Netherlands		78.1		707.1	N/A		N/A	
	North Macedonia		12.8		84.6	N/A		N/A	
	Norway		45.3		376.5	N/A		N/A	
	Poland		61.7		129.7	N/A		N/A	
	Portugal		18.6		546.8	N/A		N/A	
	Russia		22.4		124.2	N/A		N/A	
	Serbia		17		220.8	N/A		N/A	
	Singapore		20.4		178.7	N/A		N/A	
	Slovakia		200.4		413.9	N/A		N/A	
	Slovenia		106		730.3	N/A		N/A	
	Spain		14.4		308	N/A		N/A	
	Switzerland		53.1		419.1	N/A		N/A	
	Turkey		27.7		122.6	N/A		N/A	
	Ukraine		16.8		85.5	N/A		N/A	
	United Kingdom		60.1		291.7	N/A		N/A	
	United States		22.8		245.4	N/A		N/A	
	Vietnam		18.8		20.8	N/A		N/A	
**Not in outbreak**							3.2		128.6
	Argentina		4.2		252.3	N/A		N/A	
	Armenia		2.5		114.7	N/A		N/A	
	Australia		5.5		428.3	N/A		N/A	
	Azerbaijan		5.1		69.5	N/A		N/A	
	Brazil		4.9		89	N/A		N/A	
	Brunei		2.5		116.4	N/A		N/A	
	Canada		6.4		126	N/A		N/A	
	Colombia		4.2		60.1	N/A		N/A	
	Costa Rica		1.7		144.1	N/A		N/A	
	Ecuador		3.3		52.1	N/A		N/A	
	Guatemala		1.2		19.4	N/A		N/A	
	India		0.8		22.6	N/A		N/A	
	Indonesia		0.1		13.7	N/A		N/A	
	Iran		2.8		42	N/A		N/A	
	Iraq		0.8		18.1	N/A		N/A	
	Israel		5.3		1177.3	N/A		N/A	
	Japan		0.1		74.2	N/A		N/A	
	Kazakhstan		2.6		74	N/A		N/A	
	Kuwait		0.6		147	N/A		N/A	
	Mexico		1.7		38.3	N/A		N/A	
	Moldova		7.4		110.7	N/A		N/A	
	Morocco		0.4		20.2	N/A		N/A	
	Nepal		1		29.3	N/A		N/A	
	Oman		0.2		44	N/A		N/A	
	Panama		5.7		247.9	N/A		N/A	
	Peru		4.2		152.7	N/A		N/A	
	Philippines		0.5		31.9	N/A		N/A	
	Qatar		4.9		142.4	N/A		N/A	
	Romania		8.2		156.1	N/A		N/A	
	Saudi Arabia		0.1		16.1	N/A		N/A	
	South Africa		0.6		39.5	N/A		N/A	
	South Korea		6.4		90.1	N/A		N/A	
	Suriname		4.9		169.1	N/A		N/A	
	Sweden		8.9		405.2	N/A		N/A	
	Thailand		9.2		18.7	N/A		N/A	
	Tunisia		1.3		79.8	N/A		N/A	
	Zambia		0.06		21.1	N/A		N/A	
	Zimbabwe		0.2		32.4	N/A		N/A	

^a^N/A: not applicable.

**Figure 2:**
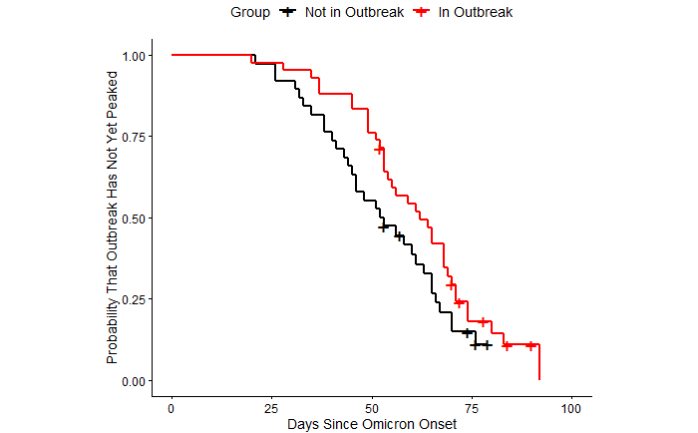
Time from arrival of Omicron until peak of outbreak. Note: Countries are considered censored if their peak speed occurred on the final day of the sample period. The cross hashes in the figure denote these censor points.

Figure 2 plots Kaplan-Meier survival curves for the 2 groups of countries [39]. An “event” was defined as the peak speed of the outbreak. We chose peak speed over the end of an outbreak because a substantial majority of sample countries remained in outbreak at the time of this writing. The survival curves present the probability a country will have reached its peak (y axis) for any given number of days since the onset of Omicron (x axis).

A key advantage of the Kaplan-Meier survival curve is its accommodation of countries that may not have hit their peak speed yet [38]. We considered any country whose peak speed occurs on the most recent day of available data “censored,” which means we only know it took at least as long as the observation period for the country to reach its peak. The Kaplan-Meier method includes these countries in its survival curve estimates until they are censored, at which point they exit the sample. The vertical hash marks in Figure 2 denote these exit points.

From Figure 2, countries already in an outbreak clearly take longer to reach their eventual peak than countries not initially in an outbreak. A log-rank test rejects the null hypothesis of equality between the 2 survival curves at the .10 significance level but not at the .05 level (*P*=.09) [51].

**Figure 3:**
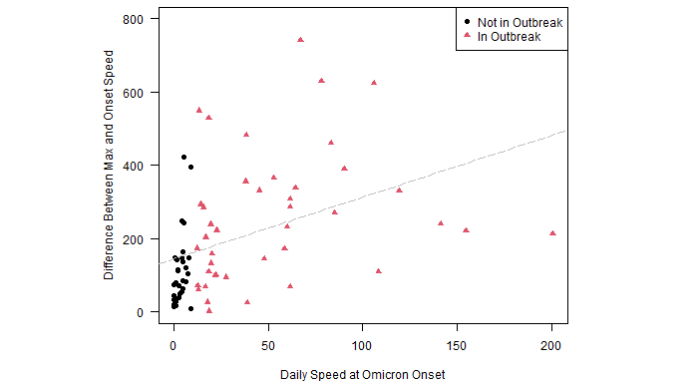
Peak of outbreak as a function of speed at Omicron arrival. Note: Not depicted, but included in the trend line calculation, is the outlier country of Israel, which reached a peak speed of 1177.3 daily novel transmissions per 100,000 population, up from a speed of 5.3 when Omicron was first sequenced in the country. The peak speed in Israel was approximately 4.5 SDs above the mean for all countries.

The survival analysis answers the question of how long the Omicron outbreak takes to peak in countries that were or were not in an outbreak at the time of Omicron’s arrival. However, the survival curves provide no information on the relative magnitudes of the peaks. To that end, Figure 3 presents a scatter plot of the difference between the eventual peak speed and the Omicron onset speed (y axis) as a function of the onset speed (x axis). The linear best fit line, in dashed gray, shows a positive association between the onset speed and the additional speed accrued after the onset of Omicron. The estimated slope coefficient is 1.62, which is statistically significant at the .01 level with a *P* value of .002.

Figure 3 plots the difference between onset and peak speed on the y axis because peak speed alone is mechanically a function of onset speed. The onset speed cannot exceed peak speed. If an outbreak immediately contracted upon the arrival of Omicron, then peak speed is simply equal to onset speed. Figure 3 therefore shows that countries that had high onset speeds at first isolation of Omicron tended to have higher peak speeds after the onset of Omicron. Higher initial speeds are correlated with higher growth after Omicron.

Figures 2 and 3 show that the Omicron-driven outbreaks took longer to build to relatively higher peak speeds in countries already experiencing outbreaks of Delta. Neither figure controls for potentially important confounders, such as population size and vaccination rates. Furthermore, neither figure controls for the length of time since the onset of Omicron. Some outbreaks may have yet to reach their apex.

To control for these confounders, Table 2 presents the results of a dynamic panel regression [41]. The model was adapted from an empirically validated system to provide novel SARS-CoV-2 surveillance metrics [35,52,53]. The dependent variable is the rate of novel SARS-CoV-2 transmissions, which is a function of transmissions on the previous day and in the past week. Unsurprisingly, these *1-day_lag* and *7-day_lag* variables are positive and statistically significant predictors of current transmissions. The coefficient estimate for *1-day_lag* is 0.1, which means, after controlling for the other covariates, every 10 SARS-CoV-2 transmissions today predict 1 transmission tomorrow. The coefficient estimate for *7-day_lag* is 0.7, which means every 1 transmission this week predicts just under 1 transmission next week.

**Table 2:** Arellano-Bond dynamic panel data estimatesa.

Variable	Coefficient (SE)	*P* value
*1-day_lag*	0.1 (0.03)	<.001
*7-day_lag*	0.7 (0.15)	<.001
*after_omi*	–62.9 (35.1)	.07
*aft_omi·in_brk*	84.1 (40.2)	.04
*days_since_omi*	0.8 (0.4)	.05
*days_since_del_outbrk*	0.4 (0.2)	.06
*weekend*	–13.5 (4.7)	.004
*total_cases_rate*	–1.1e-03 (2.2e-03)	.61
*total_vacc_rate*	1.3e-04 (9.4e-05)	.17

^a^Balanced panel: n=80, *t*=98 – 99, N=7868; Sargan test: *χ*^2^_(842)_=80 (*P*>.99); autocorrelation test 1: normal=–1.94 (*P*=.05); autocorrelation test 2: normal=–3.20 (*P*=.01).

The *after_omi* and *aft_omi·in_brk* variables provide a test for whether daily speeds during the Omicron outbreak were higher in those countries already experiencing a Delta outbreak. The former variable is an indicator set to 1 for any date after the onset of Omicron, and the latter is an interaction between *after_omi* and *in_brk*, an indicator set to 1 if a country was in a Delta outbreak at the onset of Omicron. The coefficient estimate for *aft_omi·in_brk* is 84.1 and significant at the .05 level. For the interpretation, those countries that were in a Delta outbreak saw daily speeds increase by an average of 21.2 (=84.1 – 62.9) novel transmissions per 100,000 population after the onset of Omicron.

The negative coefficient of –62.9 on the *after_omi* predictor might seem counterintuitive, but 2 factors explain the sign. First, the model controls for *days_since_omi*, which is the number of days since the onset of Omicron and the end of the sample period. The expected daily speed rises by 0.8 novel transmissions per 100,000 population for each day since the onset of Omicron, and this result is significant at the .05 level. Thus, as time passes, *days_since_omi* will eventually outweigh *after_omi*. The second factor is the tail end of Delta outbreaks. The negative effect of *after_omi* only applies to countries that were not in an outbreak at the onset of Omicron. Those countries tended to have recently exited a Delta outbreak, and *after_omi* partly captures the deceleration in speed before Omicron outbreaks gathered momentum. For the countries that were already in an outbreak at the onset of Omicron, the *days_since_del_outbrk* variable controls for how long ago the Delta outbreak began. The coefficient of 0.4 means those countries saw an average additional 0.4 transmissions per 100,000 population for each day after the Delta outbreak began. The variable is significant at the .10 level but not the .05 level.

The model also controls for weekend dates. The coefficient on the indicator variable for weekend dates, *weekend*, is negative and statistically significant at the .01 level. This result is expected because many countries fail to report complete data over weekends.

Lastly, *total_cases_rate* and *total_vacc_rate*, respectively, contain cumulative prior infections and vaccinations, as measured in rates per 100,000 population. The coefficient on *total_cases_rate* is not statistically significant at the .10 level, which is expected because prior infections offer little protection against Omicron [54]. The coefficient on *total_vacc_rate* is also not significant at the .10 level, which is expected from the considerable vaccine escape of Omicron [55]. The positive sign on the coefficient is explained by the differential vaccination rates across countries. The worst Omicron outbreaks have tended to occur in countries with higher vaccination rates. In the sample, countries already in an outbreak at the onset of Omicron had an average vaccination rate over 40% higher than the rate for countries not in an outbreak.

On a more subtle point, the technical feat of the Arellano-Bond dynamic panel is its ability to control for time-invariant, country-specific factors [41]. Examples include public health policies, demographics, population density, culture, and history. The dynamic panel estimates automatically control for these factors to the extent they remain stable over the sample period. Thus, even after controlling for vaccinations, time since the onset of Omicron, time a country had been in a Delta outbreak (if one existed at the onset of Omicron), and time-invariant, country-specific factors, the Omicron outbreak in countries with high community transmission of Delta reached larger peaks than in countries with low transmission of Delta.

### Country Comparisons

To further examine the difference between Omicron-and-Delta outbreaks and Omicron outbreaks, we compared the outbreak trajectory of several neighbor countries, at least 1 of which was in a Delta outbreak at the onset of Omicron and 1 of which was not.

**Figure 4:**
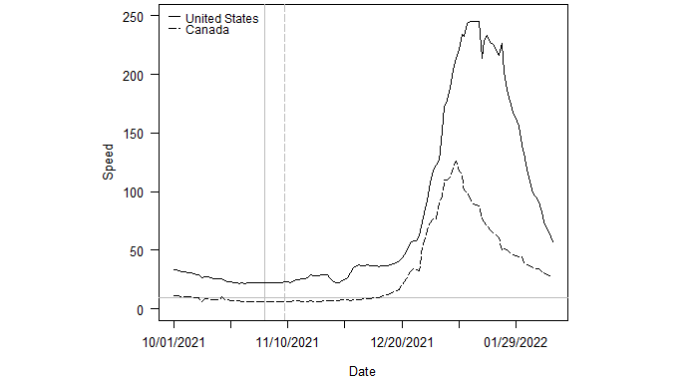
Outbreaks in Canada and the United States. Note: The vertical lines indicate the date Omicron was first sequenced in each country. The solid and dashed lines correspond to those of each country in the legend.

Figure 4 plots the rate of novel SARS-CoV-2 transmissions per 100,000 population for Canada and the United States over the sample period. The vertical gray lines indicate the date Omicron was first sequenced in each country (dashed for Canada, solid for the United States). The horizontal gray line depicts the CDC outbreak threshold for reference. A country is in a state of outbreak when its speed exceeds 10 cases per day per 100,000 population. At the onset of Omicron, Canada was not in an outbreak but the United States was.

When Omicron was first sequenced in Canada, the country had a speed of 6.4, while the speed for the United States was 22.8 at the onset date. The subsequent peak speeds for the countries were 126 and 245.4, respectively. The United States took longer to reach its peak from the date Omicron arrived, and its peak was nearly twice as high as Canada’s.

**Figure 5:**
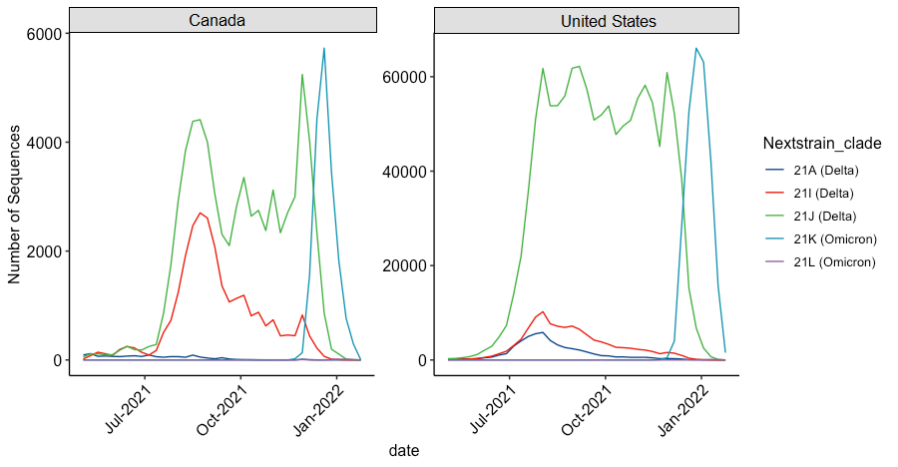
The evolution of Delta and Omicron VOCs in Canada and the United States. Note: The y axis denotes the total number of sequences for each VOC on a given date. VOCs other than Delta and Omicron were too infrequent to depict on the plot. VOC: variant of concern.

Figure 5 plots the sequencing results of SARS-CoV-2 samples from Canada and the United States from May 2021 until February 2022. Over this period, the Delta and Omicron VOCs were the primary contributors to outbreaks in both countries. The United States sequenced 10 times as many SARS-CoV-2 samples as Canada, but both countries follow roughly similar trends.

In the late summer and early fall of 2021, predominantly Delta clades were identified as part of the viral pool of SARS-CoV-2 cases in both Canada and the United States. Canada had a minor Delta outbreak, where the daily number of Delta cases slightly surpassed 10 per 100,000 population between September 15, 2021, and October 7, 2021. Canada was well below the threshold of an outbreak when Omicron was first sequenced. In contrast, the United States went into an outbreak largely driven by the Delta variant on July 19, 2021, and remained in an outbreak through the Omicron peak. Canada later went into an outbreak, largely driven by Omicron, in December, but the magnitude was roughly half that of the US outbreak. Although both countries are now only reporting sporadic new Delta cases, Delta overlapped with Omicron for the majority of the Omicron outbreak that began in December 2021. Canada cleared its Delta outbreak before the United States and before the advent of Omicron.

**Figure 6:**
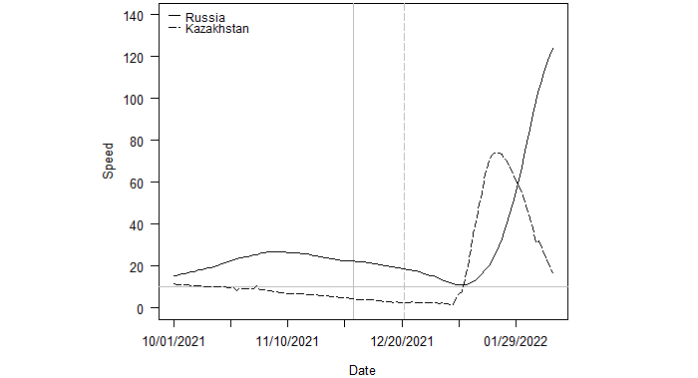
Outbreaks in Armenia, Azerbaijan, and Georgia. Note: The vertical lines indicate the date Omicron was first sequenced in each country. The solid and dashed lines correspond to those of each country in the legend.

Figure 6 provides a similar illustration for Armenia, Azerbaijan, and Georgia. Neither Armenia nor Azerbaijan was in a state of outbreak when Omicron was first sequenced in the countries, but Georgia was. The subsequent peak in Georgia was 543.7, far larger than the peaks of 114.7 and 69.5, respectively, in Armenia and Azerbaijan. Although the outbreak in Azerbaijan continues to grow, the recent decrease in acceleration indicates the country is near its apex.

**Figure 7:**
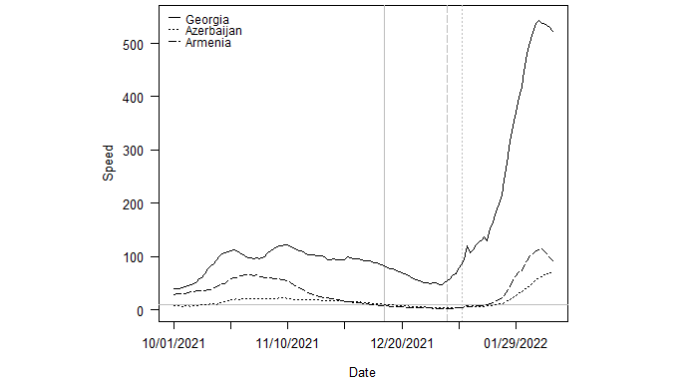
Outbreaks in Kazakhstan and Russia. Note: The vertical lines indicate the date Omicron was first sequenced in each country. The solid and dashed lines correspond to those of each country in the legend.

Figure 7 provides a similar plot for Kazakhstan and Russia. Kazakhstan was not in an outbreak at the onset of Omicron, but neighbor Russia was. The peak in Russia was 124.2, and the peak in Kazakhstan was 74.0. Russia continues to see an escalation in new transmissions, but the recent decrease in acceleration indicates the country is near its apex.

**Figure 8:**
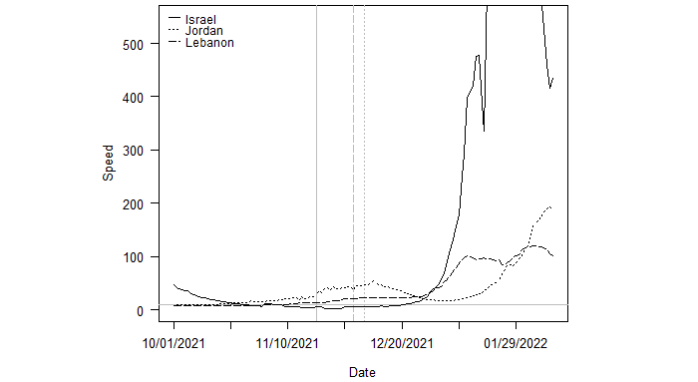
Outbreaks in Israel, Jordan, and Lebanon. Note: The vertical lines indicate the date Omicron was first sequenced in each country. The solid, dashed, and dotted lines correspond to those of each country in the legend.

Taken together, Figures 4-7 support the broader findings in Figures 2 and 3 that countries already in an outbreak at the time of Omicron’s arrival had longer durations and reached higher peaks in cases compared to countries where community transmission of Delta was already low. However, this pattern does not always hold. Israel is the most extreme exception. Despite not beginning in an outbreak, the country reached a peak speed of 1177.3 novel transmissions per 100,000 population, as shown in Figure 8. Still, these specific country illustrations provide a context and guidance for a discussion of why outbreaks in countries already in a Delta outbreak at the onset of Omicron had different trajectories than outbreaks in countries with low initially community transmission.

## Discussion

### Principal Findings

In this study, we measured the trajectory of the pandemic for every country, beginning on the first day that Omicron was sequenced, and we compared the magnitude and speed of the subsequent outbreak in countries that had high versus low levels of preexisting Delta transmission. These countries were determined to be in an outbreak or not in an outbreak at the time of their first reported Omicron sequence based on a threshold of 10 daily new SARS-CoV-2 transmissions per 100,000 population.

Our analysis of epidemiological curve trajectories for countries not in an outbreak at the time of Omicron’s arrival, such as sub-Saharan African countries and India, showed these outbreaks escalate, peak, and de-escalate rapidly, ending the outbreak with a small tail [19]. In contrast, Omicron outbreaks in countries already experiencing a Delta outbreak take relatively longer to peak and attenuate. This observation holds regardless of whether the Delta outbreak peaked before Omicron was introduced or whether the Delta outbreak was still trending upward. For example, Canada had peaked before Omicron was sequenced, while the United States peaked afterward. The apex of the Omicron-driven peak was over twofold higher in countries already in a Delta-driven outbreak. The former countries reached an average apex of 308.7 daily new cases per 100,000 population, while the latter countries reached an average apex of 128.6 (see Table 1). Even after controlling for the daily speed of the pandemic when Omicron was first identified in a particular country, we find that the magnitude of Omicron outbreaks in countries not already in an outbreak is slightly less than half the magnitude of Omicron outbreaks in countries with high levels of Delta transmission.

Prior to the emergence of Omicron, the Delta VOC made up over 97% of cases worldwide, with several countries experiencing Delta-driven outbreaks at the time of Omicron’s emergence in November 2021. Omicron subsequently led to outbreaks and outcompeted Delta in every country where genomic surveillance data are available, now accounting for over 97% of cases worldwide [56]. Although it was initially thought that high levels of Delta transmission in some countries could blunt the impact of Omicron, our data strongly suggest that outbreaks reached higher peak speeds and magnitudes in countries already experiencing Delta outbreaks.

The propensity of countries already in a Delta-driven outbreak to have more intense Omicron-driven outbreaks could be explained by at least 4 overlapping (and not mutually exclusive) factors: (1) policy, (2) climate, (3) epidemiologic trends, and (4) public health infrastructure.

First, a preexisting Delta outbreak may have signaled ineffective policies that underlay epidemiological trends, which took longer to build, peak, and attenuate during the Omicron outbreak. For example, countries already in a Delta outbreak already may have had less stringent public health measures that could have resulted in the significantly higher speeds and larger peaks upon the arrival of Omicron. To explore the possibility, we calculated the average stringency index for each country over the sample period [50]. The daily index takes a value between 0 and 100, with higher scores indicating stricter national or subnational SARS-CoV-2 policy responses. The index was unavailable for Armenia, Montenegro, and North Macedonia, which left 77 (96.3%) countries for comparison. The average score for countries in a preexisting Delta outbreak was 49.4 compared to an average of 48.2 for countries not in an outbreak at the time of Omicron’s arrival. A Welch *t* test failed to reject the null hypothesis of equal means across the 2 groups (*P*=.68). Because the number of countries in each group was greater than 30, the test passes the conventional guideline for approximate convergence in the central limit theorem [57]. If a *P* value provides a roughly graded measure of strength against the null hypothesis, the value suggests that other hypotheses may be more compatible with the data [58,59]. The Pearson correlation coefficient between the average score and a binary variable for the country group was also low, at –.04, with a 95% CI of –0.26 to 0.19. The Pearson coefficient is a measure of linear dependence between variables, which makes it the appropriate choice for an examination of policy intervention as a confounder in the linear dynamic panel regressions [60,61]. To summarize, policy differences seem to have limited explanatory power for the different trajectories of the Omicron outbreaks in these countries. Notwithstanding, although the enacted policies might not differ, the willingness of each country’s population to adhere to these policies might, with “COVID fatigue” resulting in relaxed implementation.

**Figure 9:**
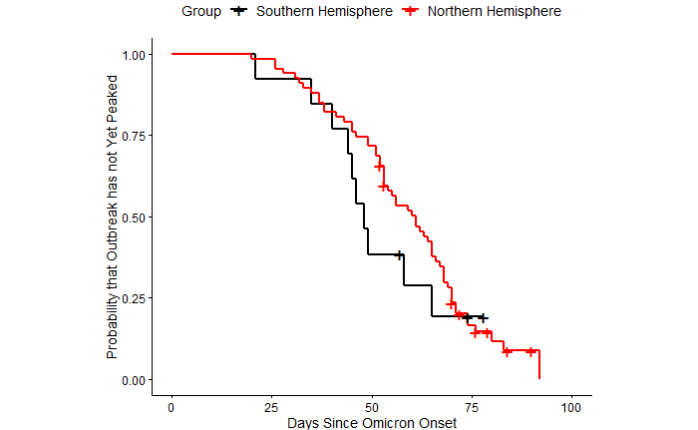
Time from arrival of Omicron until peak of outbreak by hemisphere. Note: Countries are included in the Northern Hemisphere group if their geographical area lies entirely in the Northern Hemisphere. All other countries are included in the Southern Hemisphere group.

A second explanation could be climate or socioeconomic conditions. Most countries that were still in a Delta outbreak reside in the Northern Hemisphere, and Omicron arrived over the winter months. If weather can affect the spread of SARS-CoV-2, then countries in colder regions might tend to have larger outbreaks of any variant [23-25]. Indeed, large outbreaks were observed in these countries during the winter of 2020-2021 prior to the emergence of the Delta or Omicron VOC, suggesting a seasonal trend independent of variant. Figure 9 provides survival curves analogous to those in Figure 2 but for countries that lie entirely in the Northern Hemisphere and those that do not. The curves cross at several points, which means sometimes outbreaks in the Northern Hemisphere take longer to reach their peak (from the onset date of Omicron), and sometimes outbreaks in the Southern Hemisphere do. The *P* value from a log-rank test is also higher than it was for the comparison between outbreak and nonoutbreak countries (*P*=.40 vs .09). Still, a portion of the Northern Hemisphere survival curve lies beyond the Southern Hemisphere curve, which suggests weather may partly explain why countries already in an outbreak were more adversely impacted by the arrival of Omicron. There is some visual evidence in Figure 9 that outbreaks in the Northern Hemisphere lasted longer. The Arellano-Bond method, however, controls for time-invariant, country-specific factors [41]. Climate is one such factor to the extent it remains constant for each country in the sample. In the regression estimates, temperature confounders would have to be caused by variable weather conditions over the sample period.

A third possible explanation is that the enhanced ability of Omicron to infect vaccinated individuals resulted in overlapping outbreaks with Delta in slightly different populations, increasing overall case counts. Rather than an Omicron-driven outbreak, a better description would be a Delta outbreak *and* an Omicron outbreak, at least until Delta was outcompeted. Omicron does have a higher potential for immune escape compared to prior VOCs [8,62-66]. Natural infection from SARS-CoV-2 generated a strong protection against reinfection with Alpha [67,68], Beta [67], and Delta [69], but this protection was somewhat diminished, though still robust, against Omicron [70,71]. People infected with prior VOCs remain at risk for contracting the Omicron variant [70]. Furthermore, the vaccines developed to prevent contracting SARS-CoV-2 are somewhat less effective at protection against contracting the Omicron variant [72,73]. However, although Omicron has some advantage in causing breakthrough or reinfections, it is also highly transmissible in individuals with no prior immunity, just like the Delta VOC. Therefore, it is unlikely that the 2 variants existed in independent populations and caused overlapping outbreaks. The 2 VOCs were more likely in direct competition. Omicron’s fitness advantage allowed it to quickly outcompete Delta, which is reflected in the genomic surveillance data (eg, Figure 5).

A fourth potential explanation is that the public health infrastructure in the outbreak countries better enabled them to (1) track cases in real time, (2) accurately determine the earliest date of Omicron arrival, and (3) track major surges in cases. First, given the outbreak threshold of 10 daily SARS-CoV-2 transmissions per 100,000 population, a country with better case tracking would be more likely to be in an outbreak state. Second, higher sampling for genomic surveillance is likely to result in an earlier detection of Omicron relative to the eventual peak. Third, a country with better testing infrastructure can process more tests in the context of a case surge, while other countries might be prematurely capped by capacity. Taken together, these 3 factors could explain the observed results in outbreak versus nonoutbreak countries.

Regardless of the reason for higher peaks and speeds of the Omicron outbreak in countries with preexisting Delta outbreaks, it is clear that high levels of community transmission of Delta did not substantially decrease population-level susceptibility to Omicron. First, countries in Delta outbreaks at the time of Omicron emergence still had case counts well below what would be required to elicit herd immunity. Second, even if vaccine-based immunity or natural immunity were long-lasting enough to reach herd immunity against a particular variant, an antigenic shift of SARS-CoV-2 will likely continue to drive immune evasion, as has been well documented for other RNA viruses. That being said, although Omicron resulted in many breakthrough infections and reinfections, a vast majority of these resulted in only mild disease. Thus, although case counts in many countries reached record peaks, an increasingly protected population from severe disease will likely result in a transition from a pandemic virus to an endemic virus.

### Limitations

Sequencing data are unavailable for many countries, which were not included in this study. However, enough countries remained (N=80) to statistically examine why Omicron outbreaks in countries already experiencing a Delta outbreak were significantly larger in magnitude and duration than Omicron-only outbreaks.

We also know that sequencing the index case of Omicron in each country may not capture the earliest date Omicron first arrived or a sustained transmission that led to the eventual outbreak of cases [74]. This assignment provides a proxy for when the Omicron outbreaks began. As long as the inaccuracies in the date of assignment from sequencing data are random and small, they should not cause significant bias in the dynamic panel estimates. For most countries, reassignment of the Omicron onset date causes negligible changes in the estimates.

We acknowledge that the CDC classification threshold for an outbreak is somewhat arbitrary. Small deviations from the CDC threshold rate of 10 daily SARS-CoV-2 transmissions per 100,000 population would neither reclassify most countries in the sample nor cause a significant change in estimates. Larger deviations naturally would. To address this point, we also included Figure 3 to show a broader association between the initial rate of daily SARS-CoV-2 transmissions and later peak rates, which is independent of the outbreak classification threshold.

Because we are writing this study in as close to real time as possible, the Kaplan-Meier survival curves may contain some inaccuracies. Specifically, if the peak speed occurred before the last date of the sample period, an outbreak might reverse its de-escalation in the future and reach a new, higher peak speed.

The second-order autocorrelation test for the dynamic panel estimates rejected the null hypothesis of zero autocorrelation in the unobservable error component. Although rejection in the first-order autocorrelation test is a common feature of the Arellano-Bond first-difference operation, rejection in the second-order test indicates a possible bias in coefficient estimates caused by autocorrelation in the error component [75].

Lastly, although we addressed several possible confounders, the Arellano-Bond method controls for time-invariant, country-specific variables [41]. Unobserved variations in human behavior over the sample period, for example, might remain as a source of omitted variable bias. Furthermore, the analysis of climate through hemisphere distinction is unable to capture all the nuances of local weather conditions. Temperature, wind, and humidity can all affect the spread of SARS-CoV-2 [24,25,76-78]. Likewise, the analysis of local risk factors and management capacity through the stringency index is unable to capture all the nuances of local risk and interventions [79-81].

### Comparison With Prior Work

This study builds on prior work of the Omicron VOC by Lundberg et al [19]. The original study was the first to compare singular Omicron outbreaks to previous outbreaks driven by the original SARS-CoV-2 variant, Beta, Alpha, and Delta in sub-Saharan Africa. This study compares the Omicron outbreaks in countries with high versus low community transmission of the Delta VOC at the time of Omicron’s arrival.

### Conclusion

Although it may be years before we fully understand the interplay between different SARS-CoV-2 variants, these data are likely to inform trends among groups of countries that could help predict the trajectories of future variants, given differences in preexisting case counts. Although Omicron has been emphasized as a less harmful variant, it has caused annual records of morbidity and mortality due to enhanced transmissibility and rapid spread. High community spread of Delta prior to the arrival of Omicron in some countries did not interfere with the spread of Omicron but rather portended a larger outbreak upon the arrival of the more transmissible variant.

## Abbreviations

CDC: Centers for Disease Control and Prevention
GISAID: Global Initiative on Sharing Avian Influenza Data
mRNA: messenger RNA
VOC: variant of concern
WHO: World Health Organization
